# The Defective and Dependent


**Published:** 1912-03-23

**Authors:** 


### THE DEFECTIVE AND DEPENDENT.*

### VII.—The Education of the Physically Defective.

The Education (Defective and Epileptic Children) Act of 1899 provided for the compulsory training not only of children mentally defective but also of those who, by reason of physical defect, are incapable of receiving proper instruction in the ordinary public elementary schools. In the first instance, attention was given chiefly to the education of those commonly designated "cripples," but there has been a tendency, especially since the establishment of medical inspection of schools, to extend the scope of provision for physically defective children by including other types of chronic invalids unfitted for attendance at ordinary schools, and to establish, under the powers of the Act, not only "cripple schools" but other special schools for those suffering from ophthalmia, favus, incipient pulmonary tubercle, and even chronic debility from anæmia, etc. For the two latter classes open-air schools have been tried with considerable success by the London, Bradford, Halifax, Sheffield, and other education authorities, and during the coming summer three such schools are to be available in the vicinity of London.

### Special Day-schools.

The first day-school certified for physically defectives under the Act was established in 1899 in connection with the social work of the Passmore Edwards Settlement, Tavistock Place, chiefly through the benevolent exertions of Mrs. Humphry Ward. The London School Board of that day took an active interest in this experiment, supplying the teaching staff under the supervision of Mrs. Burgwin, and encouraged by its success the system of special day-schools for physically defective children has been gradually extended throughout the metropolis, and the last published Report of the Education Officer to the London County Council gives 34 as the number of such schools, with 2,237 children in average attendance. The average cost per head is stated to be £13 2s. 6d. In relation to this comparatively high cost (about double that of the mentally defective pupil) it must be remembered that, in addition to the teaching staff, nurses and ambulance attendants have to be provided. 29 nurses and 185 other attendants (including those employed in cooking, bathing, and mid-day supervision) are engaged in the work of the 34 schools. The children are brought to school and returned to their homes in ambulances specially constructed to meet the needs of recumbent cases, and a mid-day meal is provided on the school premises, either gratuitously by the Cripple Children's Dinners Society, or upon such easy terms as the parents can afford. Special seats and tables adapted to individual cases form part of the school fittings, and room for spinal carriages, box-splints, and other surgical appliances has to be arranged for.

Following the classification of Mr. R. C. Elmslie, M.S., F.R.C.S., in an interesting paper read at the International School Hygiene Congress in London in 1907,† three types of crippled children have to be provided for in day-schools:—

(*a*) Children with tuberculous disease of the bones and joints (about 50 per cent.).

(*b*) Children with fixed deformities and paralyses (about 30 per cent.).

(*c*) Children with diseases of the heart, lungs, etc., of a chronic nature (about 20 per cent.).

### "P. D." Schools.

In the education of children so afflicted, backwardness in school attainment has to be reckoned with, due, not to mental defect, but to absence from school during long and recurring periods of treatment in hospital. To obviate this disadvantage several of the orthopædic hospitals—*e.g.* the National Orthopædic, the Alexandra Hospital for Hip Disease, etc.—have established classes for instruction of suitable patients while undergoing treatment in the wards, and such classes have been recognised by the Board of Education as "P. D." schools earning a grant under the Act. In this way the children have the advantage of appropriate surgical treatment and education simultaneously, instruction being given to many patients whilst lying in bed. Similarly the Convalescent Children's Hospital at West Kirby, near Liverpool, has been recognised as a place of instruction for the physically defective.

In view of usefulness in after-life it is, of course, most important that physical as well as educational improvement should be duly considered —country residential schools are, on that account, to be preferred to day-schools, if needful medical assistance be available, as is the case at that of the Manchester Education Committee at Swinton House, Pendlebury. A very charming residential establishment is to be found in the Cripple Colony at Chailey Downs, Sussex, associated with Mrs. C. W. Kimmins and her "Guild of the Brave Poor Things." Here are excellent schools for boys and girls respectively, registered by the Board of Education, to which the London County Council send some of their cripples to benefit by the fine air and healthy open-air life, as well as by scholastic and industrial training. A more recent Institution of similar nature is Sir William Treloar's Cripples' Home and College at Alton, Hants.

### Open-air Lessons.

The larger proportion of children classed as physically defective, owe their crippled condition to some form of tuberculosis—60 per cent. according to Dr. Elmslie—and in addition cases of

* Previous articles of this series have appeared in The Hospital of Nov. 4, Nov. 18, Dec. 2, Jan. 6, Feb. 10, and March 2.

† *I. C. S. H. Transactions,* vol. ii., p. 762-767.

incipient phthisis, of glandular affections and pronounced anæmia so often met with in slum children, are markedly benefited by an outdoor life, and apt to deteriorate under the conditions of indoor instruction. The institution of open-air schools for such cases (classed as physically defective) has consequently commended itself to the Board of Education; and special time-tables have been sanctioned prescribing *al-fresco* lessons of a practical character, *e.g.* gardening and nature study, with frequent intervals for play and recreation between book lessons of the ordinary kind. The system also provides for good nourishing meals and a post-prandial rest and sleep on deck-chairs or hammocks in the open air.

The benefits derived from the open-air schools of the London Education Committee are strikingly illustrated by photographs of children before and after their sojourn therein, which form part of Dr. Kerr's report on the subject in 1909; and details of increased weight and improved blood (augmentation of hæmoglobin) confirm this impression. Even in town schools the construction of open-air class-rooms has been attended with benefit, and in London, playground classes for instruction have been arranged experimentally during the summer months. Physically defective children have thus, owing to medical inspection, far greater consideration than formerly, with a view to preventive treatment. There are now in England and Wales sixty special schools for the physically defective, with accommodation for about four thousand children, besides seven open-air schools which provide for over six hundred.

